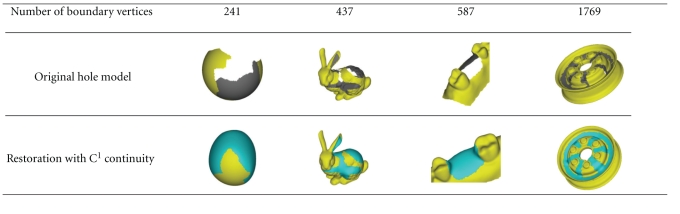# Single-Tooth Modeling for 3D Dental Model

**DOI:** 10.1155/2010/535329

**Published:** 2010-06-20

**Authors:** Tianran Yuan, Wenhe Liao, Ning Dai, Xiaosheng Cheng, Qing Yu

**Affiliations:** ^1^College of Mechanical and Electrical Engineering, Nanjing University of Aeronautics and Astronautics, Nanjing, Jiangsu 210016, China; ^2^Department of Prosthodontics, Nanjing Stomatological Hospital, Nanjing, Jiangsu 210008, China

## Abstract

An integrated single-tooth modeling scheme is proposed for the 3D dental model acquired by optical digitizers. The cores of the modeling scheme are fusion regions extraction, single tooth shape restoration, and single tooth separation. According to the “valley” shape-like characters of the fusion regions between two adjoining teeth, the regions of the 3D dental model are analyzed and classified based on the minimum curvatures of the surface. The single tooth shape is restored according to the bioinformation along the hole boundary, which is generated after the fusion region being removed. By using the extracted boundary from the blending regions between the teeth and soft tissues as reference, the teeth can be separated from the 3D dental model one by one correctly. Experimental results show that the proposed method can achieve satisfying modeling results with high-degree approximation of the real tooth and meet the requirements of clinical oral medicine.

## 1. Introduction

As the 3D (three dimensional) dental model can be acquired easily through different kinds of intra or extra oral measurement methods including optical digitizers [1–3], CT (CBCT) [4, 5] and MRI [6], CAD (Computer-Aided Design)/CAM (Computer-Aided Manufacturing) has been introduced to dentistry and achieved great success in clinical applications [7–10] such as orthodontics, oral and maxillofacial surgery. Dental restorations can be designed and manufactured much more easily compared with traditional complex and labor-intensive process. Pre- or postsurgery simulation can be used to achieve assessment of dental skeletal relationships and facial aesthetics, audit orthodontic outcomes with regard to soft and hard tissues, and direct 3D treatment planning. 

In general, the 3D dental models (including 3D single-tooth) used in CAD/CAM dentistry system are mostly obtained by optical digitizers, which is typically represented by using a watertight triangular mesh. The 3D dental model is an integral model without obvious blending boundary between the single-tooth and the soft tissues. Two adjoining teeth sometimes are fused together and without obvious tooth gap, due to teeth overlapping, lower measurement precision, and limited resolution triangulating methods during digitizing step. In order to satisfy the prerequisites of manufacturing the dental restorations and assessing the virtual dental behaviors, the teeth have to be independent of each other and keep the original shape of the real tooth. The accurate single-tooth shape restoration and extraction techniques for the 3D dental model play a vital role in CAD/CAM dentistry system. 

Although the surface of the 3D dental model is extremely irregular and complex, the fusion regions between adjoining teeth and the blending regions between teeth and soft tissues are distributed like “valleys” on the 3D dental model. So, the regions of the 3D dental model can be analyzed quantitatively by applying the corresponding geometric differential component [11, 12]—minimum curvature. The regions identified based on the geometric differential component are the clustering of vertices with similar curvature behavior, which may also include the nontarget regions. In graphics field, the target regions are usually selected by using the window polygon selection mapping method [13], which is difficult to deal with the feature regions of the 3D dental models with complex surface. In this paper, we propose a spatial polygon selection method, of which the edge is straight “line” on the 3D dental model surface.

After the fusion regions are selected and removed, the corresponding holes will be generated on the 3D dental model. Researchers have done lots of work on shape restoration for the triangle mesh models. The existing approaches can be classified into two main categories: the nongeometric [14–16] and geometric [17–21]. (1) Non-geometric methods are mainly based on the attributes of the boundary and its n-ring neighbor vertices, in order to reconstruct the field function [14, 15] or implicit surface [16] which can describe the missing part approximately. The corresponding restoration surface patch is generated by using the isosurface extraction method [22]. The restoration result of the nongeometric methods is unique, which cannot achieve restoration with given continuity, and the overall efficiency of these kinds of algorithm is low. (2) In geometric methods, the hole boundary is triangulated based on the mapping plane [20] or spatial triangulation method [18] to get an initial surface patch firstly, and then the initial surface patch is refined and reshaped to obtain the restoration surface patch. The key of the geometric methods is the triangulation of the hole boundary and the following reshaping adjustment. 

**Figure 1:**
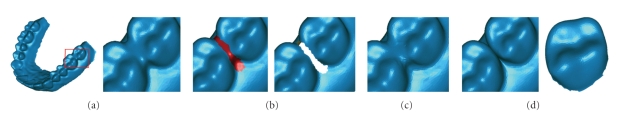
A general scheme of the single-tooth modeling.(a) Zoom view of the local region corresponding to the left first and second molars. (b) Fusion region identified and removed. (c) Restoration result with the hole filled directly (d) Restoration result that satisfies the biocharacteristics of the single-tooth.

The blending region between two adjacent teeth with obvious tooth gap is similar to a flipped “saddle” shape surface, of which the left and right sides reflect the local shape of the corresponding single-tooth, respectively. Because the surface patch reconstructed by using the existing shape restoration method represents the “whole” instead of the “partial” nature of the model, if the holes formed after the fusion regions being removed are directly filled without being further processed, we will get the restoration results similar to the original model which fails to satisfy the biocharacteristics of the single-tooth (see Figure 1(c)). In this paper, we propose a single-tooth shape restoration approach: the hole is firstly divided into two subholes and triangulated separately by using the occlusal plane as the reference; secondly, the triangulation result corresponding to each subhole is subdivided and reshaped as a whole according to the biocharacteristics of the single-tooth.

After the 3D dental model has been shape restored, the single-tooth can be extracted from the 3D dental model. Various techniques [23–25] have been proposed to segment 3D dental model, which are based on the plane view image information of the 3D dental model. The above methods are limited to segment dental models with mild malocclusion, and missed interstices or wrong cuts will be introduced when dealing with models with severe malocclusion. In this paper, we propose a segmentation boundary extraction method, which is applied directly on the 3D dental model and can separate the single-tooth from the 3D dental model correctly. 

The single-tooth modeling techniques of the 3D dental model are very important and nontrivial (see Figure 1). In this paper, we present an integrated modeling scheme, which mainly includes the following steps.

(1) Digitize the 3D dental model through extra or intra oral measurement method.
(2) Analyze, select, and remove the fusion regions between the adjacent teeth.
(3) Restore the shape of the single-tooth.
(4) Analyze and select the blending region between the teeth and soft tissues.
(5) Extract the segmentation boundary and separate the tooth from the 3D dental model.

## 2. Digital Dental Model Acquisition

Traditional measuring devices used to measure dental casts including dividers, calipers have provided the standard of plaster model analysis [26, 27], but the manual measurement techniques have disadvantages of being time consuming, inaccuracte, and capable of making linear measurements only in a few locations. With advances in computer and optical technology, the dental cast can be digitized through various scanning techniques [1–6]. The 3D dental model can benefit CAD/CAM dentistry in accuracy, efficiency, and ease of measurement of tooth size, arch form, and its dimensions.

In this paper, the 3D dental model is scanned from plaster models with a commercially available 3D scanner MCS-30 [28] depending on the structured light technique. A video camera records the structured light distortions after it has been projected over the study models, and then the computer processes the recorded images and merges them together to create a complete 3D dental model. The precision of the 3D scanner MCS-30 with 1280*1024 image resolution can reach 10 *μ*m. The average triangle numbers of the mesh that can meet the clinical precision requirement are usually no less than 20 thousands. The 3D dental model is represented by using a watertight or 2-manifold triangular mesh and usually stored as (Stereo-lithographic) STL or (Virtual Reality Modeling Language) VRML format.

## 3. Feature Regions Analysis and Extraction

### 3.1. Notation

Let *M* be the 2-manifold triangular mesh corresponding to surface *S* embedded in *R* ^3^, *V* = {*v*_1_, *v*_2_,…, *v*_*n*_} denote the set of vertices in $M\cdot {\vec{n}}_{vi}$ represents the unit normal vector of vertex *v*_*i*_. We define *N* *e* *i* *V* ^1^(*i*) as 1-ring neighbors of vertex *v*_*i*_, and get n-ring neighbors *N* *e* *i* *V* ^*n*^(*i*) through recursively enlarging the radius of the current neighborhood:

(1) $$\begin{array}{c} NeiV^{1}\left(i\right)=\left\{i\right\}+\left\{j\;|\;\exists \;\;\text{edge}\;\;\left(v_{i},v_{j}\right)\right\},\\ {NeiV}^{n}\left(i\right)=NeiV^{1}\left({NeiV}^{n-1}\left(i\right)\right)\;\left(n>1\right) \end{array}$$

*N* *e* *i* *T* ^1^(*i*) is defined as the 1-ring neighboring triangles that share vertex *v*_*i*_ · |*N* *e* *i* *V* ^1^(*i*)|, |*N* *e* *i* *T* ^1^(*i*)| denotes the set size of *N* *e* *i* *V* ^1^(*i*) and *N* *e* *i* *T* ^1^(*i*), respectively. 

### 3.2. Differential Characteristics Analysis of the 3D Dental Model

Let *p* be a point on surface *S*. Consider all curves *C* *i* on *S* passing through the point *p*. Every such *C* *i* has an associated curvature *κ*_*i*_ given at *p*. Of those curvatures *κ*_*i*_, at least one is characterized as maximal *κ*_max_ and one as minimal *κ*_min _, and these two curvatures are known as the principal curvatures of *S*. In mathematics, the minimum curvature *κ*_min_ is used to describe the hills (*κ*_min_ > 0) and valleys (*κ*_min_ < 0) of the 3D models, while the maximum curvature is used to describe ridges (*κ*_max_ > 0) and depressions (*κ*_max_ < 0).

After a detailed analysis of the 3D dental model' bioshape characteristics, we find that the blending region between the teeth and soft tissues, the fusion regions between adjoining teeth, and regions including alveolar bone ridges are distributed like “valleys” on the 3D dental model, while the regions corresponding to the incisal edges, cusp tips are “ridges” like. So, the feature regions of the 3D dental model can be classified quantitatively by using the principal curvature information. 

For the smooth triangular mesh model with uniform triangles, the second-order differential components can be solved by using the corresponding discrete differential geometry operators with guarantee accuracy, which are constructed based on the Laplace-Beltrami operator and spherical mapping methods [11]. But when the triangle shape is irregular and the mesh model is noisy, the calculation results will have much deviation compared with the real value. In this paper, we propose a local surface fitting based method used to estimate the second-order differential properties, which is proved to be robust and accurate in the following experiments. The local shape of any arbitrary complex surface can be described approximately by an *m* (*m* ≥ 2)-order polynomial surface:

(2) $$\begin{array}{c} S\left(u,v\right)=\left(u,v,\phi \left(u,v\right)\right),\\ \phi \left(u,v\right)=\sum a_{k,s}f\left(u^{k},v^{s}\right),\;0\leq k+s\leq m,\;\;k\geq 0,\;\;s\geq 0, \end{array}$$

where *a*_*k*,*s*_ is the polynomial coefficients, *f*(*u* ^*k*^, *v* ^*s*^) = *u* ^*k*^ *v* ^s^, (2) is the parameter representation of the *m*(*m* ≥ 2)-order polynomial surface in the local coordinate system. For vertex *v*_*i*_ of the mesh model, the corresponding local coordinate system *P* *u* *v* *ϕ* is determined as follows: 

Let *v*_*i*_ be the origin point of the local coordinate system. *ϕ* axis coincides with the normal ${\vec{n}}_{vi}$of vertex *v*_*i*_. *u*, *v* are orthogonal to each other in the tangents plane *T*_*i*_ of vertex *v*_*i*_. When *ϕ* axis is paralleled to *z*-axis of the absolute coordinate system *O* *x* *y* *z* after being applied for by a series of rotation and translation operation, *u*, *v* are also paralleled to *x*, *y*, respectively. 

Let *K* *N* *b*(*v*_*i*_) = {*p*_1_,…, *p*_*k*_} denote the *k* nearest neighbors of *v*_*i*_ in its n-ring neighbors *N* *e* *i* *V* ^*n*^(*i*). We apply the method proposed by Meyer et al. [11] to calculate the discrete normal vector of the triangular mesh:

(3) $${\vec{n}}_{vi}=\frac{1}{4A_{\text{mixed}}}\overset{n}{\underset{j\in NeiV^{1}\left(i\right)}{\sum }}\left(\text{cot}\;\;\alpha_{ij}+\text{cot}\;\;\beta_{ij}\right)\left(v_{i}-v_{j}\right),$$

where *α*_*ij*_ and *β*_*ij*_ are two angles opposite to the edge *e*_*ij*_ by which *v*_*i*_ and *v*_*j*_ are connected. *A*_mixed_ is the weighted summation of triangle areas from *N* *e* *i* *T* ^1^(*i*) of vertex *v*_*i*_. After ${\vec{n}}_{vi}$ is obtained, we can get the mapped vertices *K* *N* *b*(*v*_*i*_)_*Puvϕ*_ = {*q*_1_,…, *q*_*k*_} of *K* *N* *b*(*v*_*i*_) = {*p*_1_,…, *p*_*k*_} in the local coordinate system *P* *u* *v* *ϕ*, *q*_*k*_ = (*u*_*k*_, *v*_*k*_, *ϕ*_*k*_). After *P* *u* *v* *ϕ* and the vertices *K* *N* *b*(*v*_*i*_)_*Puvϕ*_ = {*q*_1_,…, *q*_*k*_} are determined, the local surface *S*(*u*, *v*) fitted to *K* *N* *b*(*v*_*i*_)_*Puvϕ*_ can be obtained by using the weighted least square method. In this paper, the corresponding least square error is

(4) $$\delta ={\underset{q_{j}\in kNb{\left(v_{i}\right)}_{Puv\phi }}{\sum }\left|\phi \left(u_{j},v_{j}\right)-\phi_{j}\right|}^{2}\cdot e^{-d_{i,j}/\max\left(d_{i,j}\right)},$$

where *d*_*i*,*j*_ = ||*q*_*i*_ − *q*_*j*_||, 1 ≤ *i*,  *j* ≤ *k*. In order to make the local surface be solved with high efficiency, *m* = 2 is applied in this paper. When *k* = 16~20, the local surface can achieve a better approximation of the real shape. According to the first and second fundamental forms of *S*(*u*, *v*), the Gaussian curvature *κ*_*G*_ and mean curvature *κ*_*H*_ at *u* = 0, *v* = 0 can be solved. Because the differential characters of the mesh model at vertex *v*_*i*_ can be substituted by the differential characters of the local surface *S*(*u*, *v*) at *u* = 0, *v* = 0, the minimum curvature *κ*_min _(*v*_*i*_) of vertex *v*_*i*_ can be calculated by the following equation:

(5) $$\kappa_{\min\;}\left(v_{i}\right)=k_{H}-\sqrt{k_{H}^{2}-k_{G}}.$$

In order to make the solved minimum curvature by (5) reflect the regional characters much more accurately, the curvature values have to be smoothed and denoised further:

(6) $$\kappa_{\min\;}\left(v_{i}\right)=\underset{j\in nei^{1}\left(i\right)}{\sum }\theta_{i,j}\kappa_{\min\;}\left(v_{j}\right),$$

where *θ*_*i*,*j*_ = 1/||*v*_*i*_ − *v*_*j*_||.

**Figure 2:**
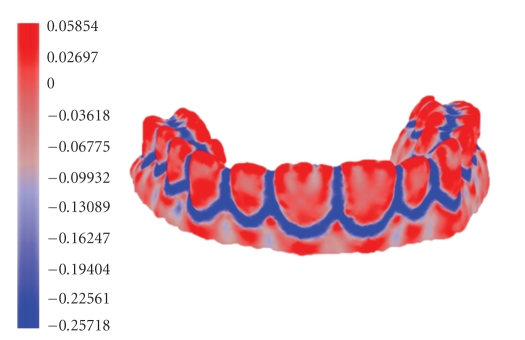
Minimum curvature color map of the 3D dental model.

We draw a color map of the minimum curvature values as shown in Figure 2 to visualize where the high- and low-curvature areas locate. The highest and lowest curvatures are corresponding to the red and blue color, respectively, the remains are assigned colors between red and green according to the curvature values. As can be seen from Figure 2, the region marked with blue can include the fusion regions and blending regions clearly. 

**Figure 3:**
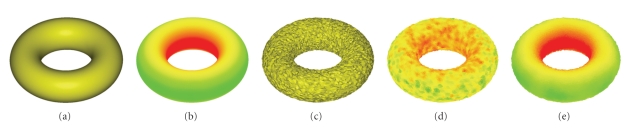
(a) Torus model with *R* = 2 and *r* = 1. (b) The accurate mean curvature plot of (a). (c) Gaussian noisy model with *h* = 0.5; (d) Mean curvature plot of (c) by Meyer et al. [11]. (e) Mean curvature plot of (c) by the method in this paper.

**Figure 4:**
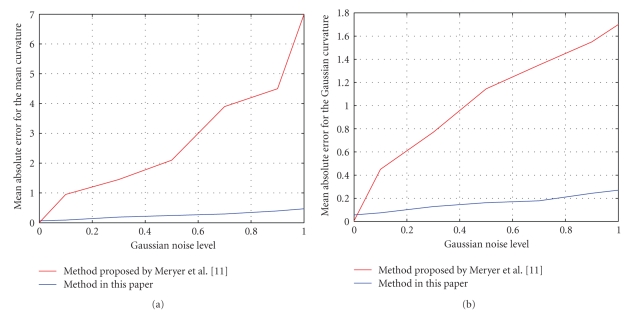
Average absolute error comparison. (a) Mean curvature. (b) Gaussian curvature.

We compare the curvature evaluation method proposed in this paper with that of Meyer et al. [11] by using the torus model:

(7) $$\begin{array}{c} r\left(u,v\right)=r\left(x\left(u,v\right),y\left(u,v\right),z\left(u,v\right)\right),\\ \begin{array}{c} x\left(u,v\right)=\left(R+r\;\cos\;\;\;v\right)\cos\;\;u \end{array},\\ y\left(u,v\right)=\left(R+r\;\cos\;\;\;v\right)\sin\;u,\;0\leq u\leq 2\pi ,\;\;0\leq v\leq 2\pi ,\\ z\left(u,v\right)=r\;\sin\;v, \end{array}$$

where *R* is the wheel radius and *r* is the tube radius. In this paper, we choose *R* = 2 and *r* = 1 as the torus parameters. We obtain the corresponding noisy torus model by adding Gaussian noise with noise level *h* = 0.1,0.3,0.5,0.7,0.9,1.0, respectively. Figure 3 shows that the mean and Gaussian curvatures are robust to noise, and the estimation results (see Figure 4) are much more stable and reliable than those of Meyer et al. [11] when the differential components of noise model are calculated by the method proposed above.

### 3.3. Post Processing of the Feature Regions

**Figure 5:**
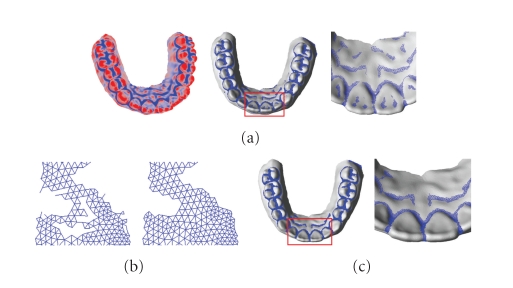
(a) Feature regions extracted corresponding to vertices marked with blue in the minimum curvature color map. (b) Feature region before and after being applied for opening and closing operation; (c) Feature regions of (a) after being processed further.

After the 3D dental model has been analyzed based on the minimum curvature information, the regions of the 3D dental model can be classified and extracted according to the given curvature threshold. However, the feature regions extracted usually contain small pieces and holes (see Figure 5(a)). The small pieces contained in the feature regions can be identified efficiently according to the vertex neighboring relationship, and can be removed from the feature regions automatically when the vertex number of the small pieces is less than the given value. We use the mathematical morphology operation extended from the image field to fill the small holes and smooth the feature regions boundaries. There are also four main operators such as *d* *i* *l* *a* *t* *i* *o* *n*, *e* *r* *o* *s* *i* *o* *n*, *o* *p* *e* *n* and *c* *l* *o* *s* *e* included in the 3D mathematical morphology operation [29]. 

Let *F*′denote the index set of the vertices in the feature regions. ∀*j* ∈ *F*′⇒*v*_*j*_ ∈ *M* = (*v*_1_,…, *v*_*i*_,…, *v*_*n*_), (1 ≤ *j* ≤ *n*), the *d* *i* *l* *a* *t* *i* *o* *n* and *e* *r* *o* *s* *i* *o* *n* morphology operators corresponding to the 3D models are defined as follows:

(8) $$\begin{array}{c} {\text{dilation}}^{n}\left(F^{\prime }\right)=\left\{k\;|\;\exists j\in F^{\prime }:k\in NeiV^{n}\left(j\right)\right\},\\ {\text{erosion}}^{n}\left(F^{\prime }\right)=\left\{k\;|\;NeiV^{n}\left(k\right)\in F^{\prime }\right\}. \end{array}$$

Dilation operation is used to “attract” the vertices unmarked as feature vertices but lying inside or at the boundary of the feature regions and can still keep the “shape” of the feature region during dilating. Erosion operation is used to delete undesired branches and will make the feature regions seem much more smooth and thin. We obtain the opening operation by consecutively dilating and then eroding the feature region. The closing operation is obtained by swapping the applying order:

(9) $$\begin{array}{c} {\text{open}}^{n}\left(F^{\prime }\right)={\text{erosion}}^{n}\left(F^{\prime }\right)\circ {\text{dilation}}^{n}\left(F^{\prime }\right),\\ {\text{close}}^{n}\left(F^{\prime }\right)={\text{dilation}}^{n}\left(F^{\prime }\right)\circ {\text{erosion}}^{n}\left(F^{\prime }\right). \end{array}$$

Multiple application of opening and closing operation can filter out the noise and artifacts of the feature regions effectively. Figure 5(b) shows the feature region after being applied for opening and closing operation. 

### 3.4. Spatial Polygon Selection Method

As can be seen from Figure 5(c), after being further processed, the feature regions *F*′ can include the fusion regions between the adjacent teeth and the blending regions between the teeth and the soft tissues completely. However, because the feature regions extracted according to the given threshold are the clustering of the vertices with similar curvature behavior, the nontarget regions such as areas including alveolar bone ridges are also extracted. In order to obtain the target regions accurately, the feature regions have to be divided and selected interactively. In this paper, we propose a spatial polygon selection method, of which the edge is straight “line” on the 3D dental model surface.

**Figure 6:**
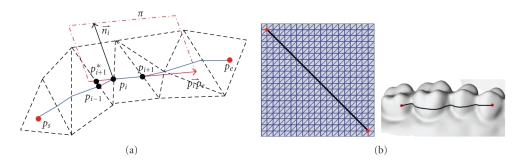
(a) Concept of the direction tracing method (b) Examples of “line” solved on the planar and spatial surface.

Let *p*_*s*_ and *p*_*e*_ denote the starting position and the destination on the triangular mesh *M*. The spatial “line” *α* = (*p*_*s*_, *p*_1_ ⋯ *p*_*n*_, *p*_*e*_) between these two vertices on the triangular mesh model can be solved approximately by using the direction tracing method described as follows:

Let$\vec{n_{i}}$, $\vec{n_{i+1}}$be the normal of *p*_*i*_, *p*_*i*+1_. Assume *Q* with unit length is the object being moved on the surface of the triangular mesh. If *p*_*i*_ is the current position, and *p*_*i*+1_ is the next position *Q* going along the direction $\vec{p_{i}p_{e}}$ as shown in Figure 6(a), we do not change the direction $\vec{p_{i}p_{e}}$and make sure that the pose of *Q* parallels to the normal $\vec{n_{i}}$ when *Q* is moving in the interior of a triangle or along an edge until it get to *p*_*i*+1_. When *Q* is at *p*_*i*+1_, we change the moving direction $\vec{p_{i}p_{e}}$into$\vec{p_{i+1}p_{e}}$, and the pose $\vec{n_{i}}$ into $\vec{n_{i+1}}$. Because (*p*_0_,…, *p*_*i*−1_, *p*_*i*_, *p*_*i*+1_,…, *p*_*n*_) is piecewise linear continuous and lies over the triangular mesh, *p*_*i*_ and *p*_*i*+1_ must belong to the same triangle. The line segment$\vec{p_{i}p_{i+1}}$ is also the intersection between the triangle which includes *p*_*i*_,*p*_*i*+1_ and the normal section *π* at *p*_*i*_ through $\vec{p_{i}p_{e}}$. We can obtain *p*_*i*+1_ according to *p*_*i*_, *p*_*e*_ and $\vec{n_{i}}$as shown in Figure 6(a). The incident triangles of *p*_*i*_ intersecting with the normal section *π* may be more than one sometime. Let *p*_*i*+1_^1^, *p*_*i*+1_^2^⋯ denote the intersection points. We choose *p*_*i*+1_* as *p*_*i*+1_ when the angle between $\vec{p_{i}p_{i+1}^{*}}$ and $\vec{p_{i}p_{e}}$ is the smallest of all the *p*_*i*+1_* ∈ (*p*_*i*+1_^1^, *p*_*i*+1_^2^⋯). Beginning with *p*_*s*_, *p*_1_, *p*_2_ ⋯ *p*_*i*_ ⋯ is solved in turn. When *p*_*i*_ and *p*_*e*_ are in the same triangle, we get the 3D “line” *α* = (*p*_*s*_, *p*_1_ ⋯ *p*_*n*_, *p*_*e*_) (see Figure 6).

**Figure 7:**
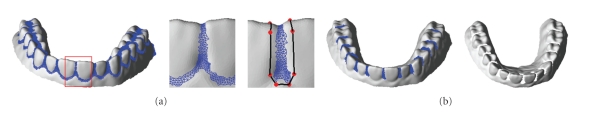
(a) Fusion region extraction example by using the spatial polygon selection method; (b) Results of the fusion regions being extracted and removed.

As shown in Figure 7(a), the edges of the spatial polygon can be determined by a series of vertices on the 3D model, which are selected interactively according to the profile of the target region. The triangles are marked with “select”, of which the three vertices fall into the inner of the spatial polygon together.

## 4. Single Tooth Shape Restoration

After the fusion regions have been removed, the corresponding holes are generated on the 3D dental model. The holes are typical “saddle shape” and each one is shared by two adjoining single-teeth (see Figure 1(b)). If the holes are directly filled without being further processed, we will get the restored model similar to the original (see Figure 1(c)). The failure reason is that the hole belongs to two teeth which are adjoining but independent from each other. If the hole is filled as a whole, the boundary information of the two independent teeth will be diffused into the same restoration surface patch averagely, and cannot reflect the biocharacteristics of the single-tooth. In this paper, we present a single-tooth shape restoration approach. In order to achieve a best approximation of the original tooth, the restoration process has to satisfy the following prerequisites.

- The restoration surface patch corresponding to the missing part should be reconstructed in a way that is minimally distinguishable from the surrounding regions, and should also preserve the sampling density of the original 3D dental model.
- There is no interference between the restored teeth according to the independence properties of the teeth. The blending region between the adjacent teeth has to be natural and continuous.

Let *B* denote the hole boundary of the 3D dental model. *P* represents the filling patch for *B*. *P* ^min ^, *P* ^refine^ and  *P* ^deform^ are the surface patches corresponding to different filling stages: spanning triangulation, refinement, deformation. *P* ^final^ represents the final restoration result, which meets the restoration prerequisites.

### 4.1. Hole Boundary Triangulation

In geometric methods, the hole boundary is mostly triangulated based on the mapping plane [20] or spatial triangulation method [18] to get an initial surface patch. The mapping plane triangulation methods convert the 3D hole boundary into the 2D polygon by projecting it onto the mapping plane, which is fitted to the boundary vertices by the least square method. The mapping plane triangulation methods can achieve satisfying results in dealing with the simple regular hole, which is homeomorphic to a disc after projection. But for the complex hole with sharp curvature changes along the boundary, there will appear self-intersection in the projected 2D polygon. Barequet and Sharir [18] give an interesting solution of the 3D polygon triangulating problem. The spatial triangulation method [18] has order of *O*(*N* ^3^) time complexity (*N* is number of the boundary vertices), which is adaptable to deal with the boundary with small vertices number, but difficult to triangulate the big hole. In this paper, we proposed a spatial triangulation method based on local optimized weight rule, in which various influencing factors that may affect the triangulation are considered completely.

We define Ω : *B* ^3^ → *L* as the weight function, where *B* = {*v*_1_^*b*^, *v*_2_^*b*^,…, *v*_*n*_^*b*^}, *L* is the weight set and Ω assigns a weight for each triangle with three consecutive vertices of *B*. Let *A*(*v*_*i*_) denote the sum of the adjacent angles of the current vertex *v*_*i*_

(10) $$A\left(v_{i}\right)=\overset{}{\underset{j\in NeiT^{1}\left(i\right)}{\sum }}A_{j}.$$

**Figure 8:**
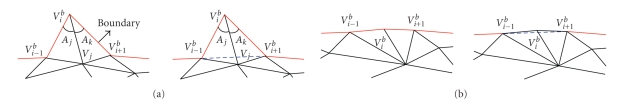
Abnormal results of the triangle being added when *A*(*v*_*i*_^*b*^) < *α* *π*. (a) Form sharp corner; (b) Generate triangle with interior angle close to *π*.

During the triangulation process, when 0 < *A*(*v*_*i*_^*b*^) < *α* *π*, after the new triangle (*v*_*i*−1_^*b*^, *v*_*i*_^*b*^, *v*_*i*+1_^*b*^) is added as shown in Figure 8, sharp corners or triangle with interior angle close to *π* will be formed. In order to avoid such situations to appear, the candidate triangle (*v*_*i*−1_^*b*^, *v*_*i*_^*b*^, *v*_*i*+1_^*b*^) should be assigned a weight *l*_less_ with lower choice priority when 0 < *A*(*v*_*i*_^*b*^) < *α* *π*. we found empirically that *α* = 1.2 can yield good results. 

In the 2-manifold triangular mesh model, the better number of neighboring triangles is usually 5 to 8. In order to avoid too many new generated triangles converge at the same boundary vertex, the number of the vertex 1-ring neighboring triangles has to be limited during triangulation. So, when |*N* *e* *i* *T* ^1^(*i*)| > 8, the vertex *v*_*i*_^*b*^ should be removed firstly, which means that the candidate triangle (*v*_*i*−1_^*b*^, *v*_*i*_^*b*^, *v*_*i*+1_^*b*^) should be assigned a weight *l*_bigger_ with higher choice priority. At the same time, when the current vertex' 1-ring neighboring triangles are projected onto their own tangent plane, there should be no intersection between the projected edges except at the current vertex itself. So, the new added triangle (*v*_*i*−1_^*b*^, *v*_*i*_^*b*^, *v*_*i*+1_^*b*^) has to satisfy the nonintersection projection condition at *v*_*i*−1_^*b*^,*v*_*i*_^*b*^,*v*_*i*+1_^*b*^ simultaneously.

When |*N* *e* *i* *T* ^1^(*i*)| ≤ 8, *A*(*v*_*i*_^*b*^) > *α* *π*, the weight of the candidate triangle (*v*_*i*−1_^*b*^, *v*_*i*_^*b*^, *v*_*i*+1_^*b*^) should be determined by its own geometric attributes such as edge length, area, and interior angle. In order to obtain a triangulation surface patch with moderate internal changes, the edges should be distributed along the boundary averagely similar to a curtain covering at a window, and the vertices of the edges should be the pairs relative nearest to each other in space. So, the candidate triangle (*v*_*i*−1_^*b*^, *v*_*i*_^*b*^, *v*_*i*+1_^*b*^) should be weighted according to its corresponding edge length. The smaller the perimeter of the triangle is, the higher choice priority it will have. 

Based on the analysis of the influencing factor which will affect the triangulation results, weight functions Ω, *l*_less_, and *l*_bigger_ are described as follows:

(11) $$\begin{array}{cc} & \Omega \left(v_{j}^{b},v_{i}^{b},v_{k}^{b}\right)\\ & =\{\begin{array}{cc} -\left(||e_{ij}||+||e_{ik}||+||e_{jk}||\right) & \text{when}\left|NeiT^{1}\left(i\right)\right|\leq 8,\\ & \;\;\;\;\;A\left(v_{i}^{b}\right)>\alpha \pi ,\\ l_{\text{less}} & \text{when}0<A\left(v_{i}^{b}\right)<\alpha \pi ,\\ & \;\;\;\;\;\left|NeiT^{1}\left(i\right)\right|\leq 8,\\ l_{\text{bigger}} & \text{when}\left|NeiT^{1}\left(i\right)\right|>8,\\ -\infty & \text{when}\;\;\text{intersection}\;\;\\ & \;\;\;\;\;\text{after}\;\;\text{projection}, \end{array} \end{array}$$

where *l*_less_(*v*_*j*_^*b*^, *v*_*i*_^*b*^, *v*_*k*_^*b*^) = −(*π*/*A*_sum_)*R_*C*_, *l*_bigger_(*v*_*j*_^*b*^, *v*_*i*_^*b*^, *v*_*k*_^*b*^) = (|*N* *e* *i* *T* ^1^(*i*)|/8)*R_*C*_, and *R*_*C*_ is the radius of the model' bounding sphere. We apply the following procedure to implement the triangulation process:

(12) $$v_{n+1}^{b}=v_{1}^{b},\;\;v_{n+2}^{b}=v_{2}^{b}$$

**Step 1**

Compute all the weights Ω(*v*_*i*−1_^*b*^, *v*_*i*_^*b*^, *v*_*i*+1_^*b*^) according to the weight function given above for each triangle (*v*_*i*−1_^*b*^, *v*_*i*_^*b*^, *v*_*i*+1_^*b*^) with three consecutive vertices of *B*, and insert the weights into *L* in which the weight is sorted using an AVL tree.

**Step 2**

Select the maximum *l*_max_ from the weight set *L*, and insert its corresponding triangle (*v*_*i*−1_^*b*^, *v*_*i*_^*b*^, *v*_*i*+1_^*b*^) into *M*_*C*_. Remove the weights of the triangles Ω(*v*_*i*−2_^*b*^, *v*_*i*−1_^*b*^, *v*_*i*_^*b*^), Ω(*v*_*i*−1_^*b*^, *v*_*i*_^*b*^, *v*_*i*+1_^*b*^) and Ω(*v*_*i*_^*b*^, *v*_*i*+1_^*b*^, *v*_*i*+2_^*b*^) from *L* that include vertex *v*_*i*_^*b*^. Eliminate vertex *v*_*i*_^*b*^ from *B*, and then *B*_*H*_ = {*v*_1_^*b*^, *v*_2_^*b*^,…, *v*_*i*−2_^*b*^, *v*_*i*−1_^*b*^, *v*_*i*+1_^*b*^, *v*_*i*+2_^*b*^,…, *v*_*n*_^*b*^}. Compute the weights Ω(*v*_*i*−2_^*b*^, *v*_*i*−1_^*b*^, *v*_*i*+1_^*b*^), Ω(*v*_*i*−2_^*b*^, *v*_*i*−1_^*b*^, *v*_*i*+1_^*b*^) of the triangle (*v*_*i*−2_^*b*^, *v*_*i*−1_^*b*^, *v*_*i*+1_^*b*^), (*v*_*i*−1_^*b*^, *v*_*i*+1_^*b*^, *v*_*i*+2_^*b*^), and insert them into *L*.

**Step 3**

Execute Step 2 iteratively until the vertex number of *B* is less than three, and obtain the initial surface patch *P* ^min^.

### 4.2. Subhole Division

**Figure 9:**
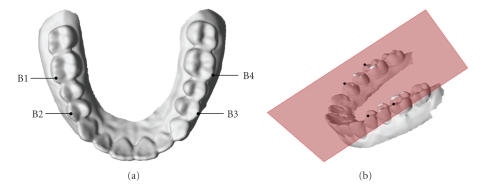
(a) B1, B2, B3, and B4 are the corresponding reference points. (b) The occlusal plane fitted to the four reference points.

In order to reconstruct the surface patch with the shape of the flip “saddle”, the hole boundary *B* has to be divided into two subholes *B* ^1^, *B* ^2^ as shown in Figure 11(a). Each subhole is corresponding to its own tooth. The end points of the bridge edge, by which the hole boundary is bridged to form two separate subholes, are two points farthest to the occlusal plane on the buccal and lingual side of the hole boundary respectively, and can be selected automatically by using the occlusal plane as the reference. In this paper, the occlusal plane is fitted with four reference points (including the buccal cusp tips of the left and right first molars, and the mesiobuccal points of the left and right first permanent molars) as shown in Figure 9.

The subhole *B* ^1^ (*B* ^2^) is first filled in with a local optimized triangulation *P*_1_^min ^ (*P*_2_^min ^) of its 3D contour (see Figure 11(b)). The initial subfilling surface patches *P*_1_^min ^, *P*_2_^min ^ combine a complete initial filling surface patch *P* ^min ^ for *B* together (see Figure 11(c)). 

### 4.3. Refinement

**Figure 10:**
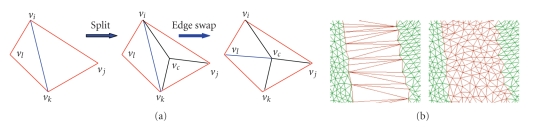
(a) Refinement and optimization mechanism. (b) Surface patch before and after being refined.

Because the edges in the initial surface patch *P* ^min ^ are the direct connections between boundary vertices, the surface patch *P* ^min ^ has to be refined according to the boundary information to obtain a further surface patch *P* ^refine^, which approximates the density of the surrounding mesh. The mesh density is usually measured based on the average length of the edges. In this paper, the bigger triangle (*v*_*i*_, *v*_*j*_, *v*_*k*_) is split into three smaller ones by using “1-3” face splitting method, in which the new added vertex is the centroid *v*_*c*_ = (*v*_*i*_ + *v*_*j*_ + *v*_*k*_)/3 of the triangle (*v*_*i*_, *v*_*j*_, *v*_*k*_), and the interior edges are relaxed while splitting to maintain a Delaunay-like triangulation (see Figure 10).

### 4.4. Reshaping

*P* ^refine^ is still a surface patch with *C* ^0^ continuity both at the boundary and the internal. The surface patch *P* ^refine^ has to be reshaped in order to generate a surface patch, which can both reflect the local characteristics of the missing part and have a good degree of visual reality. In this paper, we design a reshaping adjustment scheme based on the discrete Euler-Lagrange equation. The reshaping adjustment scheme is described as follows. 

Let *S* : Ω → *R* ^3^ be the smooth surface corresponding to *M*. *S*_∗_ denotes the k-order partial derivatives, and *δ*Ω stands for the surface boundary. The quadratic energy function [30] for the surface is

(13) $$E_{k}\left(S\right)=\int F_{k}\left(S_{u\cdots u},S_{u\cdots uv},\ldots ,S_{v\cdots v}\right).$$

In order to actually compute the solution to the above optimization problem, we apply variational calculus to derive the corresponding Euler-Lagrange equation which characterizes the minimizers of (13)

(14) $$\begin{array}{c} \Delta^{k}S\left(x\right)=0,\;x\in \Omega \setminus \delta \Omega \\ \Delta^{j}S\left(x\right)=b_{j}\left(x\right),\;x\in \delta \Omega ,\;\;j<k, \end{array}$$

where Δ is the Laplace Operator, *b*_*j*_ (*j* < *k*) is the boundary constraints. In order to ensure the efficiency and stability of algorithms, the value range of *k* are limited to 1 ≤ *k* ≤ 3. When we use a triangle mesh as the underlying surface representation, the Laplace operator is discretized as

(15) $$\Delta \left(v_{i}\right)=\frac{2}{\text{Area}\left(v_{i}\right)}\overset{}{\underset{j\in NeiV^{1}\left(i\right)}{\sum }}\left(\text{cot}\;\;\alpha_{ij}+\text{cot}\;\;\beta_{ij}\right)\left(v_{i}-v_{j}\right),$$

where Area(*v*_*i*_) is the area sum of the vertex' 1-ring neighboring triangles, and *α*_*ij*_ and *β*_*ij*_ are two angles opposite to the edge *e*_*ij*_. The higher order Laplace operator can be solved iteratively

(16) $${\bar{\Delta }}^{k}\left(v\right)=\Delta \left({\bar{\Delta }}^{k-1}\left(v\right)\right).$$

And then, (14) becomes a linear equation with sparse matrix

(17) $$\left[\frac{{\bar{\Delta }}^{k}}{0\;|\;I_{F}}\right]\left(\begin{array}{c} P\\ F \end{array}\right)=\left(\begin{array}{c} 0\\ F \end{array}\right),$$

where *P* = (*v*_1_,…, *v*_*p*_) are the free vertices interior of the surface patch. *F* = (*f*_1_,…, *f*_*F*_) are the constraint vertices with *C* ^*k*−1^ boundary continuity. For *k* = 1, *k* = 2, and *k* = 3, the surface solved from (17) is corresponding to a membrane with minimization surface area, a thin plate with minimization bending, and a surface with minimization curvature variation, respectively. 

According to the geometric characteristics of the tooth surface, the surface patch obtained after being reshaped should be a surface with minimum bending variation. So, the constraint parameter *k* is assigned the value 2 in this paper. During the deformation stage, the triangles that have greater shape change should be refined again. If we apply *P* ^deform^ as the final result *P* ^final^ directly, small interference will appear between the adjoining teeth sometimes (see Figure 12). We use the following equation to control the deformation degree:

(18) $$P^{\text{final}}=P^{\text{refine}}+\lambda \left(P^{\text{deform}}-P^{\text{refine}}\right),\;0<\lambda \leq 1.$$

Figure 12 shows the restoration results with *λ* assigned different values. The value of *λ* determines the deformation degree of the restoration patch. 

## 5. Single Tooth Extraction

After the 3D dental model has been shape restored, the single-tooth can be extracted from the 3D dental model. The differential information of the 3D dental model is re-analyzed and processed by using the methods proposed above. As can be seen from Figure 14(a), the feature regions identified based on the minimum curvature value can include the blending regions completely, and has already possessed the coarse profile of the segmentation boundary. The feature regions are still too coarse to be accepted as the segmentation boundary. We have to peel the vertices of the region boundary inward until obtaining its skeleton with width of one vertex. The key step of the boundary extraction is how to judge a vertex should be peeled or not, and the skeleton must follow the original topology of the feature region. In this paper, the segmentation boundary extraction procedure is designed based on the vertex complexity [29].

∀*i* ∈ *F*′; let *C* *n* *e* *i*(*i*) denote the 1-ring neighborhood of vertex *v*_*i*_ ordered counter clockwise. ∀*k* ∈ *C* *n* *e* *i*(*i*); if *k* ∈ *F*′ at the same time, we record *C* *n* *e* *i*(*i*)_*k*_ = 1 or *C* *n* *e* *i*(*i*)_*k*_ = 0. With the above assumption, the vertex complexity CP(*i*) of *v*_*i*_ is defined as follows (see Figure 13):

(19) $$\text{CP}\left(i\right)=\overset{k\leq m}{\underset{k=1}{\sum }}\left|Cnei{\left(i\right)}_{k}-Cnei{\left(i\right)}_{k+1}\right|.$$

If CP(*i*) ≥ 4, vertex *v*_*i*_ is defined to be complex. If *C* *n* *e* *i*(*i*)⊆*F*′, vertex *v*_*i*_ is defined as center vertex. The neighbor of the center vertex is called satellite vertex, when its corresponding complexity is no less than zero. During the boundary extracting, the center vertex and complex vertex are marked as feature vertices that should be preserved. If the center vertex is removed, small close ring will be formed in the inner of the feature region, and the regional connectivity will be undermined if the complex vertex is removed. The set of satellite vertices is denoted by *F*_*S*_, center vertices by *F*_*C*_, and complex vertices by *F*_CP_. Then, we obtain the set of candidate vertices *F*_*D*_ that will be removed as follows.

(20) $$F_{D}=F_{S}\cap \bar{F_{\text{CP}}\cup F_{C}}.$$

We remove one vertex from the candidate set *F*_*D*_ each time, and recalculate its neighboring vertex' complexity simultaneously. The set *F*_*S*_, *F*_*C*_, *F*_CP_ and *F*_*D*_ are updated after each removing. The removing and updating operation is iterated until the “shape” of the feature regions does not change anymore. 

The skeleton obtained after being applied for the above operation also contains unnecessary open branches as shown in Figure 14(b). Because the segmentation boundary used to extract the single-tooth is a set of closed rings. The branches can be identified and pruned by deleting the line segment from the skeleton iteratively, which has at least one endpoints only connected with itself. Sometimes, there will be small redundant close rings existing, which is need to be removed interactively. After pruning, we obtain the segmentation boundary as shown in Figure 14(c). Figure 14(d) shows the single-tooth extracted according to the segmentation boundary. 

## 6. Experimental Results and Analysis

In order to verify the validity and adaptability of the proposed method, we have conducted a series of experiments on various types of 3D models. Figures 16 and 17 show the modeling results of the two typical kinds of 3D dental models (see Table 3) including model with normal tooth arrangement and model with severe malocclusion. Table 1 shows the detailed information of the 3D dental models including bounding box size, vertex/triangle numbers before and after shape modeling. 

As can be seen from Figure 2, the minimum curvature calculation method proposed in this paper can detect the fusion regions effectively. After the 3D dental model has been analyzed quantitatively based on the minimum curvature, and processed further by applying the morphology operation, we can extract the target regions according to the corresponding regional characteristics (see Figure 7(b)). 

Figure 6 shows that the spatial “line” solved by the direction tracing method proposed is an approximate geodesic curve, which has a linear time complexity of *O*(*n*), where *n* is the vertex number of the “line”. The polygon selection is real-time, and the time consumed can be omitted. So, the target regions can be selected fast and intuitively (see Figure 7). 

When we use the weight rule proposed in this paper to triangulate the hole boundary, at the initial stage, because the number of the neighboring triangles is small, the boundary is triangulated primarily based on the adjacent angles and the perimeter of the candidate triangle. As shown in Figure 18(b), the initial stage is also a process used to eliminate the saw tooth and smooth the boundary. As the boundary is triangulated continuously, the weight rule will select a vertex at the corner with the highest choice priority as the forwarding location. The two vertices of the new added edge usually have much higher choice priority than the rest of the boundary, which will drive the triangulation forward until a curtain like surface patch is covering at the boundary (see Figures 18(c) and 18(d)). The weight rule divides the triangulation process into boundary smoothing and boundary zipping approximately, by which a uniform and natural triangulation surface patch can be obtained.The time complexity of the proposed method is *O*(*N* log *N*) (N is the number of the boundary vertices).

**Figure 11:**
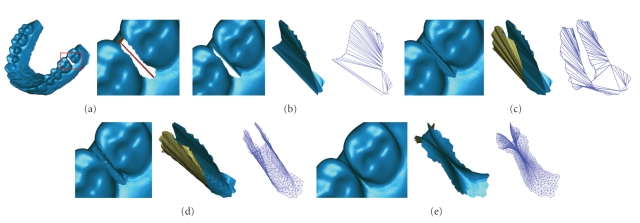
(a) Hole bridged to divide into two separate subholes. (b) Triangulation of the subhole. The corresponding shaded and wireframe surface patches. (c) After completely filling of the entire hole. (d) After refinement. (e) After reshaping adjustment.

**Figure 12:**
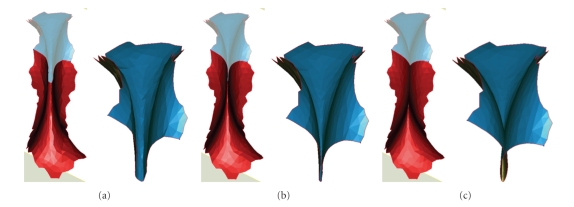
The values of *λ* determines the deformation degree of the restoration patch. From left to right: the refined mesh patch deformed with *λ* = 0.7, *λ* = 0.85, and *λ* = 1.0.

**Figure 13:**
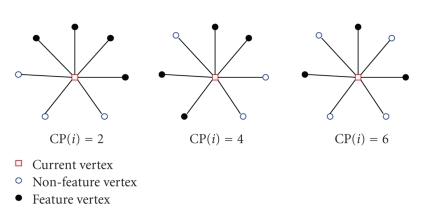
Examples of vertex' complexity computation.

As can be seen from Figures 10 and 11, the refinement surface patch can achieve a similar mesh density with the original model, which can avoid the situation of irregular triangles to appear when the surface patch is applied by the reshaping adjustment operation. During the reshaping adjustment stage, in order to control the deformation degree, the parameter *λ* was introduced in (18) to ensure the restored surface patch satisfies both the continuality and noninterference conditions. We apply the method proposed by Park [31] to detect the self-intersection. The parameter value of *λ* is limited to the range from 0.8 to 1.0 based on a great deal of experimental analysis, and the adjustment step *τ* should not be bigger than 0.01. Then, the deformation degree can be adjusted from *λ* = 1 to *λ* = 1 − k*τ automatically until there is no intersection existing. We apply the incremental least squares method [32] to solve the reshaping matrix, which can reach the rate of 50000 vertices per seconds on the personal computer with P4, 2.4 GHz processor.

We compared the triangulation quality (see Figure 15) and efficiency (see Table 2) with the method proposed by Barequet and Sharir [18]. As can be seen from Figure 15, the method proposed in this paper can deal with complex holes with much more uniform triangulation result than that of Barequet and Sharir [18]. The triangulation efficiency of [18] is measured in minutes, and it takes no less than half an hour to deal with the 13–15 holes of the 3D dental model (without single-tooth missing), which cannot meet the actual efficiency needs. Statistical results of Table 2 show that the average triangulation and restoration efficiency can reach 20000 V/s and 4000 V/s, respectively by using the method proposed in this paper. The vertex number of the restoration surface patch is usually 1/30~1/40 of the original 3D dental model. So, the whole shape modeling procedure of the 3D dental model can be complete in 2~3 minutes.

**Figure 14:**
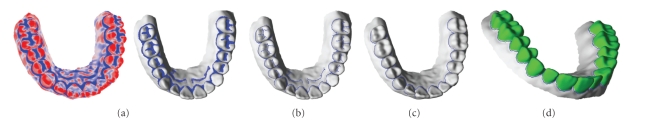
Procedure of segmentation boundary extraction and single-tooth separation. (a) 3D dental model with extracted feature regions. (b) after peeling. (c) after pruning. (d) single-tooth separation.

**Figure 15:**
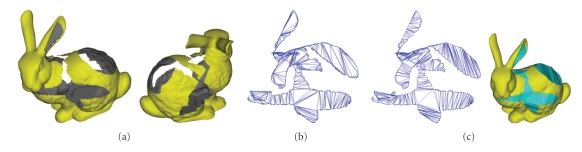
Triangulation results comparison. (a) Bunny model with complex hole. (b) Triangulation result by Barequet and Sharir [18]. (c) Triangulation result by the method in this paper.

Figure 14 shows that the blending regions between the teeth and the soft tissues can be extracted completely. Because the skeleton of the nontarget regions such as areas including alveolar bone ridges is open branches, which can be removed automatically in the pruning stage, the nontarget regions donot need to be removed interactively. The regions can be used to the extract segmentation boundary directly. 

**Figure 16:**
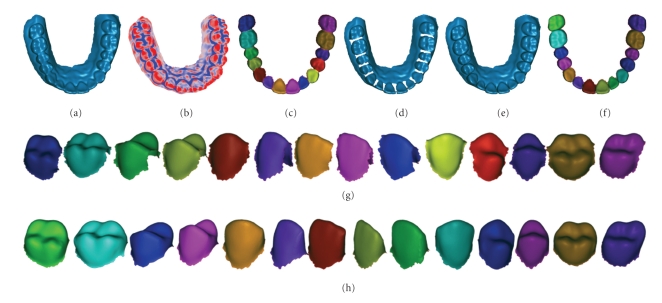
Single-tooth modeling results of 3D dental model with normal tooth arrangement. (a) The initial 3D dental model. (b) Minimum curvature color map. (c) Separation results shown as a whole corresponding to the initial 3D dental model. (d) After the fusion regions being deleted. (e) After single-tooth shape being restored. (f) Separation results corresponding to the 3D dental model after shape restoration. (g) The separated tooth in (c) displayed, respectively. (h) The separated tooth in (f) displayed, respectively.

**Figure 17:**
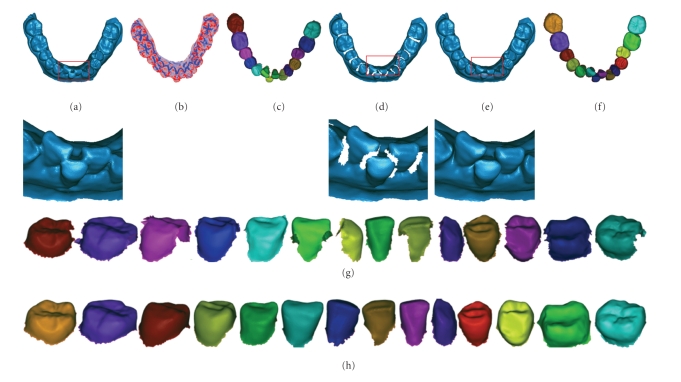
Single-tooth Modeling results of 3D dental model with severe malocclusion. (a)The initial 3D dental model. (b) Minimum curvature color map. (c) Separation results shown as a whole corresponding to the initial 3D dental model. (d) After the fusion regions being deleted. (e) After single-tooth shape being restored. (f) Separation results corresponding to the 3D dental model after shape restoration. (g) The separated tooth in (c) displayed, respectively. (h) The separated tooth in (f) displayed, respectively.

**Figure 18:**
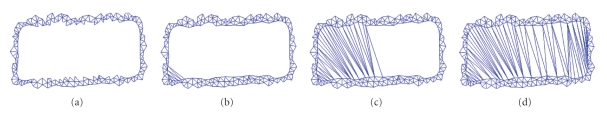
Triangulation mechanism. (a) Initial hole. (b) Eliminating saw tooth and smooth the boundary. (c) Closing the hole in the zipping way. (d) The triangulation results.

The separated teeth in Figures 16 and 17 show that the modeling techniques proposed in this paper can restore the shape of the single-tooth and segment the teeth correctly. 

In order to test the accuracy, we have done lots of experiments on comparing the restored tooth with its corresponding plaster single-tooth. Statistical results show that the radial deviation between this two models is usually ranging from 0 to 50 um, which can meet the clinical requirements.

## 7. Conclusion

In this paper, we proposed an integrated single-tooth modeling scheme, which is mainly composed of fusion regions extraction, single-tooth shape restoration and separation. As can be seen from the above examples, the modeling results can satisfy the biocharacteristics of the real tooth. Unlike the method based on plan-view range image of teeth, we directly compute bioinformation needed on the 3D dental model. We have demonstrated that the modeling scheme can achieve satisfying modeling results with high degree approximation of the original tooth and meet the requirements of clinical oral medicine.

## Figures and Tables

**Table 1:** The detailed information of the 3D dental models.

	Bounding box size (unit: mm)	Vertex (V)/Triangle numbers (T)	
		Before shape modeling	After shape modeling
Model in Figure 16	68.3*38.1*50.05	131508 (V)/262140 (T)	148020 (V)/295164 (T)
Model in Figure 17	67.4*28*49.8	145548 (V)/290116(T)	186849 (V)/372608 (T)

**Table 2:** Time consumption and number of vertices (V)/triangles (T) generated at different stages in shape restoration with *C* ^1^ continuity.

Number of boundary vertices	Generate *P* ^min ^		Generate *P* ^refine^	Generate *P* ^final^
	*O* (*N* ^3^) time complexity of Barequet and Sharir [18]	*O*(*N* log *N*) time complexity of method in this paper	(including time consumed during generating *P* ^min ^)	(including time consumed during generating *P* ^refine^)
241	91.40s (241V/239T)	0.015s (241V/239T)	0.297s (4581V/8826T)	1.005s (5264V/10182T)
437	191.844s (437V/435T)	0.016s (437V/435T)	0.392s (6031V/11636T)	1.232s (6844V/12962T)
587	1408.14s (857V/855T)	0.031s (857V/855T)	0.173s (2663V/4628T)	0.677s (2733V/4828T)
1769	12762.3s (1769V/1767T)	0.062s (1769V/1767T)	0.328s (4559V/7342T)	1.104s (4743V/7986T)

**Table 3:** Models corresponding to different boundaries in Table 2 before and after shape restoration.